# A note regarding the mathematical treatment of a class of steady‐state compartmental models of the circulation

**DOI:** 10.14814/phy2.12945

**Published:** 2016-09-01

**Authors:** Ronald J. White

**Affiliations:** ^1^ Montana Tech Butte Montana

**Keywords:** cardiovascular system, closed circulation, intrathoracic pressure, steady‐state compartmental model

## Abstract

A class of steady‐state compartmental models of the circulation is examined and it is shown that the mathematical problem for this model class involves a single nonlinear equation. In an important subclass and with certain assumptions regarding the form of the Starling‐type cardiac function curves, the single equation is of the form *Z* = *μ* + *λ* log[(1 − *Z*)/*Z*] where *μ* and *λ* are mathematical parameters related to the physiological parameters of the system and *Z* is proportional to the cardiac output. This result holds regardless of the number and arrangement of compartments within the model itself or of the number of physiological parameters the model contains. An example of this class with 25 physiological parameters is presented to illustrate this approach.

## Introduction

Compartmental models of the cardiovascular system have been useful for many years, going back to seminal work by Guyton et al. (1972, 1973). Modern versions of such compartmental models have continued to contribute to our understanding of physiology today (Thomas et al. 2008; Kofránek and Rusz 2010; Hester et al. 2011; Moss et al. 2012; Artiles et al. 2016). Although most of today's compartmental models are dynamic, time‐independent steady‐state models have played an important historical role in cardiovascular modeling, and this paper examines the mathematical structure obtained in an idealized class of compartmental models representing a steady‐flow state within a closed circulation. The resulting equations illustrate the fact that the underlying mathematical nature of the solution of this system is independent of the number and arrangement of the compartments involved. For a special subclass of such models, it is shown that the required solution depends only on a single nonlinear equation with a small number of mathematical parameters. This approach is applied to a previously studied (Coleman et al. 1974) five‐compartment cardiovascular model with parallel visceral and peripheral compartments, but with added variable intrathoracic and abdominal pressures.

## General Model Description

The general circulatory model examined in this paper has N total compartments, with M pulmonary compartments. It is assumed that: the model is in a steady state; the circulation is closed; flow is nonpulsatile and flow out from each side of the heart is described by a Starling‐type cardiac function curve; flow out from the noncardiac compartments depends linearly on the pressure difference between the upstream and downstream compartments; linear compliance curves relate pressure and volume in each compartment; and no venous collapse occurs near the right heart.

The following conventions are adopted:

- Compartment 1 represents the right atrium and it will be designated by “R” or “1” interchangeably. It is considered as a part of the systemic circulation. Note that, in this formulation, right atrial pressure (*P*_R_ = *P*_1_) is equal to central venous pressure (CVP).
- Compartments 2 to M + 1 are compartments within the pulmonary circulation, with compartment M + 1 representing the left atrium, designated by “L” or “M + 1” interchangeably.
- Compartments M + 2 to N are the remaining compartments within the systemic circulation.
- Compartmental volume (*V*_*n*_) is related to compartmental pressure (*P*_*n*_) by the following linear relation: $V_{n}=V_{n}^{0}+C_{n}\times \left(P_{n}-P_{n}^{e}\right)$ where *C*_*n*_ is the compartmental compliance, $V_{n}^{0}$ is the compartmental unstressed volume, and $P_{n}^{e}$ denotes any compartmental external pressure.
- Except for the right and left atria, compartments have arterial and venous resistances at the inflow and outflow ends, respectively, designated by RA_*n*_ and RV_*n*_.
- Flow out from the right and left atria are assumed to be determined by the following nonlinear parameterization of the Starling‐type cardiac function curve (Guyton et al. 1973; White et al. 1983):

(1) $$F_{R}=\frac{K_{R}}{1+\alpha_{R}\times \exp\left[-\beta_{R}\times \left(P_{R}-P_{R}^{e}\right)\right]},\;\text{and}\;F_{L}=\frac{K_{L}}{1+\alpha_{L}\times \exp\left[-\beta_{L}\times \left(P_{L}-P_{L}^{e}\right)\right]}$$

where *F* represents ventricular output, and *K*,* α*, and *β* are heart‐specific parameters.

Several immediate conclusions result from these assumptions. First, and most importantly, an immediate consequence of the closed circulation is the fact that blood volume is constant in this class of models. This simple conservation law leads to a key relationship valid for all such models whether in a steady state or not. This primary conservation law states

(2) $$\text{BV}=V_{0}+\sum_{n=1}^{N}V_{n}=\text{constant}$$

where BV is the blood volume and *V*_0_ is the volume of blood in the noncapacitive (noncompartmental) regions of the circulation (and not contributing to the pressure within the system). Thus,

(3) $$\text{BV}=V_{0}+\sum_{n=1}^{N}V_{n}^{0}+\sum_{n=1}^{N}C_{n}\times P_{n}-\sum_{n=1}^{N}C_{n}\times P_{n}^{e}.$$

In the steady state, the flow out from the right and left sides of the heart (*F*_R_ and *F*_L_) are both equal to the cardiac output, *Q*, and the total flows through both the pulmonary circulation and the systemic circulation are also equal to *Q*. It follows (White et al. 1983) that all of the compartmental pressures can be written as:

(4) $$\begin{array}{cc} P_{n}= & P_{L}+G_{n}\times Q\;\text{if}\;n=\text{pulmonary system compartment},\\ & \text{and}\\ P_{n}= & P_{R}+G_{n}\times Q\;\text{if}\;n=\text{systemic system compartment} \end{array}$$

where the *G*_*n*_ are functions of the resistances in either the pulmonary or systemic circulation and depend on the particular structure (number of compartments and their arrangement) selected for the circulatory system model.

Equation (3), the conservation law, may be rewritten in the following form:

(5) $$C_{S}\times P_{R}+C_{P}\times P_{L}+\text{CR}\times Q=\Delta \text{BV}+\sum_{n=1}^{N}C_{n}\times P_{n}^{e}=D\;\left(\text{constant}\right)$$

where

$$\begin{array}{cc} C_{S} & =C_{1}+\sum_{n=M+2}^{N}C_{n},\\ & C_{P}=\sum_{n=2}^{M+1}C_{n},\\ & \text{CR}=\sum_{n=2}^{M}C_{n}\times G_{n}+\sum_{n=M+2}^{N}C_{n}\times G_{n} \end{array}$$

and

$$\Delta \text{BV}=\text{BV}-V_{0}-\sum_{n=1}^{N}V_{n}^{0}.$$

Equation (5) is the equation for a plane in the coordinates (*P*_R_, *P*_L_, *Q*). This plane will be called the “conservation plane” since it results from the application of the conservation of blood to the general model. Note that this equation is independent of the form assumed for the Starling‐type cardiac function curves, equation (1).

For the steady‐state circulatory system model, equations (1) and (5) represent three equations in three unknowns and, in principle, may be solved for the three unknowns: cardiac output, and right and left atrial pressures. From these quantities, all other variables may be obtained. Three general approaches for solving these equations will be discussed and illustrated using a five‐compartment model example.

The first approach involves utilizing a *three‐dimensional graphical analysis*. By plotting each of the three relationships using the coordinate system (*P*_R_, *P*_L_, *Q*), one can find their single mutual intersection and thus the unique steady‐state model solution. As noted above, the three equations represent a plane and two “sigmoidal waves” in this coordinate system. It is interesting to note that most of the model parameters of physiological significance, outside of the heart itself, are folded into the equation describing the conservation plane. Thus, if the heart parameters do not change, the physiological variables (cardiac output, compartmental pressures, and volumes) are all determined by “movement” of the conservation plane in the three‐dimensional space as the various noncardiac parameters change.

The second approach involves utilizing a *two‐dimensional graphical analysis* that results from eliminating one of the atrial pressures using the two cardiac function relationships, equation (1), in the steady state. For example, by setting the flow from both sides of the heart to the same value, one can derive the following relation:

(6) $$P_{L}=P_{L}^{e}-\frac{\beta_{R}}{\beta_{L}}\times P_{R}^{e}+\frac{1}{\beta_{L}}\times \log\left[\frac{\alpha_{L}}{\alpha_{R}}\right]+\frac{\beta_{R}}{\beta_{L}}\times P_{R}.$$

Combining this result with equation (5) yields the relation

(7) $$Q=\frac{D-C_{P}\times \left(P_{L}^{e}-\frac{\beta_{R}}{\beta_{L}}\times P_{R}^{e}+\frac{1}{\beta_{L}}\times \log\left[\frac{\alpha_{L}}{\alpha_{R}}\right]\right)-\left(C_{S}+C_{P}\times \frac{\beta_{R}}{\beta_{L}}\right)\times P_{R}}{\text{CR}}=A-B\times P_{R}$$

where *A* and *B* are constants. If this linear function, termed as the “composite flow curve” for convenience, and the right heart function *F*_R_ = *Q* are both plotted in the (*P*_R_, *Q*) coordinate system, the intersection of the two functions defines the solution of the steady‐state model. Note that this plot has the appearance of the intersection of a sigmoidal cardiac output curve with a linear “venous return” curve (Beard and Feigl 2011).

The third approach involves reducing the three relations among *P*_R_, *P*_L_, and *Q* to a *single nonlinear equation* by eliminating *P*_R_ and *P*_L_ using equation (1), and substituting the result into equation (5). This yields the following single equation for *Q*, the steady‐state cardiac output:

(8) $$\text{CR}\times Q=D-C_{S}\times P_{R}^{e}-C_{P}\times P_{L}^{e}+\log\left[{\left(\frac{K_{R}-Q}{\alpha_{R}\times Q}\right)}^{\frac{C_{S}}{\beta_{R}}}\times {\left(\frac{K_{L}-Q}{\alpha_{L}\times Q}\right)}^{\frac{C_{P}}{\beta_{L}}}\right].$$

This equation has only five mathematical parameters regardless of the number and arrangement of the compartments. This is best seen when the above equation is written in the form:

(9) $$Q=\gamma_{1}+\gamma_{2}\log\left[\frac{{\left(K_{R}-Q\right)}^{\gamma_{3}}{\left(K_{L}-Q\right)}^{1-\gamma_{3}}}{Q}\right]$$

where *γ*_1_, *γ*_2_, and *γ*_3_ are constants. No exact solution to this equation in terms of known functions has been found. However, numerical solutions are readily obtainable since convergence is stable and rapid.

## Special Case: Right/Left Heart Balance

In a special case where the two sides of the heart have the same maximum pumping capability, *K*_R_ = *K*_L_ = *K*, and where both sides of the heart are subjected to the same external pressure, $P_{R}^{e}=P_{L}^{e}=P^{e}$, equations (8) or (9) can be simplified further. In this case, it is convenient to introduce the dimensionless variable *Z* = *Q*/*K*, the fraction of maximum cardiac output, resulting in the following general equation of the circulation for this special class of models:

(10) $$Z=\mu +\lambda \times \log\left(\frac{1-Z}{Z}\right),$$

where *μ* and *λ* are dimensionless parameters defined by

(11) $$\mu =\frac{D-\left(C_{S}+C_{P}\right)\times P^{e}-\log\left(\alpha_{R}^{\frac{C_{S}}{\beta_{R}}}\times \alpha_{L}^{\frac{C_{P}}{\beta_{L}}}\right)}{K\times \text{CR}},$$

and

(12) $$\lambda =\frac{\frac{C_{S}}{\beta_{R}}+\frac{C_{P}}{\beta_{L}}}{K\times \text{CR}}.$$

Equation (10) holds for the physiological region 0 < *Z* < 1, since the cardiac output must lie between 0 and the maximum value (*K*). Note that *Z* is uniquely determined by the values of the parameters *μ* and *λ*, but that many different values of the two parameters may lead to the same value of *Z*.

Using the Lagrange inversion theorem (Weisstein; Whittaker and Watson 1990) to solve equation (10), it leads to a formal series solution in the parameter *λ*:

(13) $$Z\left(\mu ,\lambda \right)=\mu +\sum_{n=1}^{\infty }\frac{\lambda^{n}}{n!}\frac{d^{n-1}}{d\mu^{n-1}}\left\{\log^{n}\left(\frac{1-\mu }{\mu }\right)\right\}.$$

whose first four terms are:

(14) $$Z\left(\mu ,\lambda \right)=\mu +\lambda \log\left[\frac{1-\mu }{\mu }\right]\left(1-\frac{\lambda }{\mu (1-\mu )}+\frac{\lambda^{2}}{\mu^{2}{\left(1-\mu \right)}^{2}}\right)+\frac{\lambda^{3}\left(1-2\mu \right)}{2\mu^{2}{\left(1-\mu \right)}^{2}}\log^{2}\left[\frac{1-\mu }{\mu }\right]+O\left(\lambda^{4}\right)$$

## Example: A Five‐Compartment Model

**Figure 1:**
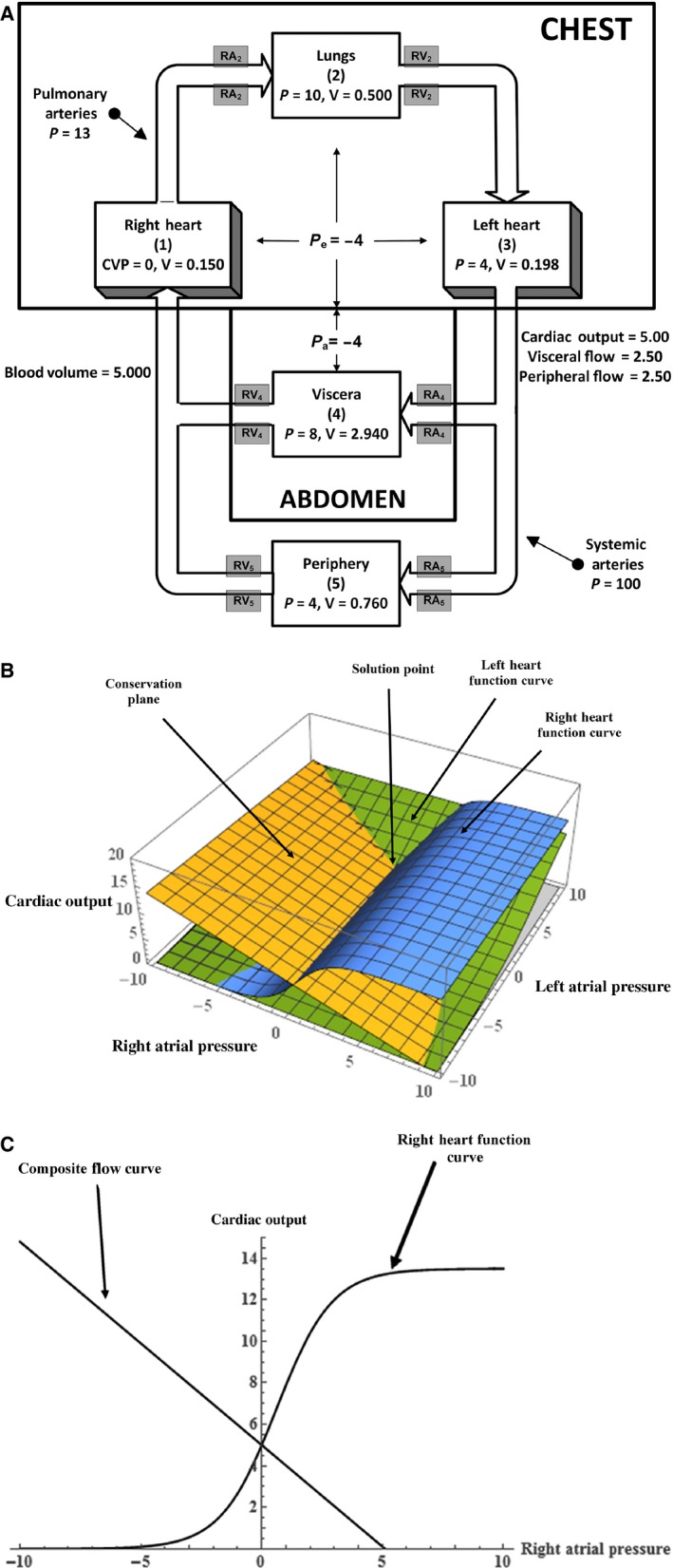
(A) The normal steady state of the five‐compartment model of the circulation with chest and abdominal cavities, as described in the text. (See Table 2.) The units for the variables shown in these figures are: flow – L/min, pressure – mmHg, volume – L. (B) Three‐dimensional graphical analysis of the normal model steady state showing intersection of the right and left heart Starling curves and the conservation plane. (C) Two‐dimensional graphical analysis of the normal model steady state showing the intersection of the right heart Starling curve and the composite flow (“venous return”) curve.

To illustrate the previous approach with a concrete example, the general formalism developed above will be applied to the five‐compartment model of the circulation whose normal state is illustrated in Figure 1A. This model is nearly identical to a previously well‐studied model (Coleman et al. 1974), except that it includes chest and abdominal compartments and uses the Starling curves from equation (1). The chest compartment surrounds the heart and lung compartments with the intrathoracic pressure in the chest assumed to be uniform and equal to *P*_e_, that is, $P_{R}^{e}=P_{L}^{e}=P_{e}$. The abdominal compartment surrounds the visceral compartment with a uniform pressure equal to $P_{4}^{e}=P_{a}$. In the steady state, *P*_a_ = *P*_e_ (Agostoni and Rahn 1960). In this model, external pressure on the peripheral bed is ignored. For simplicity, this example assumes *K*_R_ = *K*_L_ = *K*. Table 1 provides the parameter values used for the model in the normal steady state, while Table 2 provides the values of the mathematical and physiological variables in that state.

**Table 1:** Normal values assumed for the parameters used in the five‐compartment circulatory model. The cardiac function curve parameters were taken from (White et al. 1983), the intrathoracic and abdominal pressures were taken from (Agostoni and Rahn 1960), and the remaining parameters were taken from (Coleman et al. 1974)

Parameter type	Parameter	Value
Volume (L)	BV – blood volume	5.000
	*V*_0_ – blood volume in non‐capacitive regions	0.452
	$V_{1}^{0}$ – unstressed volume of the right atrium	0.102
	$V_{2}^{0}$ – unstressed volume of the lungs	0.290
	$V_{3}^{0}$ – unstressed volume of the left atrium	0.102
	$V_{4}^{0}$ – unstressed volume of the viscera	1.380
	$V_{5}^{0}$ – unstressed volume of the periphery	0.600
Compliance (L/mmHg)	C_1_ – compliance of the right atrium	0.012
	C_2_ – compliance of the lungs	0.015
	C_3_ – compliance of the left atrium	0.012
	C_4_ – compliance of the viscera	0.130
	C_5_ – compliance of the periphery	0.040
Resistance (mmHg/L/min)	RA_2_ – arterial resistance of the lungs	0.6
	RV_2_ – venous resistance of the lungs	1.2
	RA_4_ – arterial resistance of the viscera	36.8
	RV_4_ – venous resistance of the viscera	3.2
	RA_5_ – arterial resistance of the periphery	38.4
	RV_5_ – venous resistance of the periphery	1.6
External pressure (mmHg)	*P*_e_ – intrathoracic (chest) pressure	−4
	*P*_a_ – abdominal pressure	−4
Cardiac parameters	*K* – maximum value of cardiac output (L/min)	13.5
	*α*_R_ – parameter used in sigmoidal flow equation for right heart (see eq. (2)), dimensionless	55.2
	*β*_R_ – parameter used in sigmoidal flow equation for right heart (see eq. (2)) (/mmHg)	0.870
	*α*_L_ – parameter used in sigmoidal flow equation for left heart (see eq. (3)), dimensionless	23.1
	*β*_L_ – parameter used in sigmoidal flow equation for left heart (see eq. (3)) (/mmHg)	0.326

**Table 2:** Steady‐state values of the physiological variables in the five‐compartment model of the circulation. Units used: flow – L/min, pressure – mmHg, volume – L, resistance – mmHg/L/min. All pressures are measured relative to normal atmospheric pressure (760 mmHg)

Variable	Name	Equation	Normal value	Case 1 resistance changes	Case 2 external pressure changes
*μ*	Mu	See equation (17)	0.326	0.536	0.372
*λ*	Lambda	$\frac{\frac{C_{S}}{\beta_{R}}+\frac{C_{P}}{\beta_{L}}}{K\times \text{CR}}$	0.0838	0.138	0.0838
*Z*	Scaled cardiac output	$\mu +\lambda \times \log\left(\frac{1-Z}{Z}\right)$	0.370	0.523	0.404
*Q*	Cardiac output	KZ	5	7.1	5.5
*P*_1_ = *P*_R_ = CVP	Right atrial pressure (also central venous pressure)	$P_{e}+\log{\left(\frac{\alpha_{R}Q}{K-Q}\right)}^{\frac{1}{\beta_{R}}}$	0	0.70	−3.8
*P*_2_	Pulmonary pressure	*P*_L_ + RV_2_ *Q*	10	14	7
*P*_L_ = *P*_3_	Left atrial pressure	$P_{e}+\log\;\;{\left(\frac{\alpha_{L}Q}{K-Q}\right)}^{\frac{1}{\beta_{L}}}$	4	5.9	0.4
*P*_4_	Visceral pressure	*P*_R_ + RV_4_ *f*_4_ *Q*	8	5.5	4.9
*P*_5_	Peripheral pressure	*P*_R_ + RV_5_ *f*_5_ *Q*	4	9.6	0.5
PA	Arterial pressure	*P*_R_ + TPR*Q*	100	116	105
PPA	Pulmonary arterial pressure	*P*_L_ + (RA_2_ + RV_2_)*Q*	13	19	10
*V*_1_	Right atrial volume	$V_{1}^{0}+C_{1}\left(P_{1}-P_{e}\right)$	0.15	0.16	0.15
*V*_2_	Pulmonary volume	$V_{2}^{0}+C_{2}\left(P_{2}-P_{e}\right)$	0.50	0.56	0.52
*V*_3_	Left atrial volume	$V_{3}^{0}+C_{3}\left(P_{3}-P_{e}\right)$	0.20	0.22	0.20
*V*_4_	Visceral volume	$V_{4}^{0}+C_{4}\left(P_{4}-P_{a}\right)$	2.94	2.62	3.06
*V*_5_	Peripheral volume	$V_{5}^{0}+C_{5}P_{5}$	0.76	0.98	0.62
TPR	Total peripheral resistance	$\frac{\left({\text{RA}}_{4}+{\text{RV}}_{4}\right)\left({\text{RA}}_{5}+{\text{RV}}_{5}\right)}{{\text{RA}}_{4}+{\text{RV}}_{4}+{\text{RA}}_{5}+{\text{RV}}_{5}}$	20	16	20
*f*_4_	Fraction visceral flow	$\frac{\text{TPR}}{{\text{RA}}_{4}+{\text{RV}}_{4}}$	0.50	0.21	0.50
*f*_5_	Fraction peripheral flow	1 − *f*_4_	0.50	0.79	0.50

With these assumptions, the equation defining the *conservation plane*, equation (5), becomes

(15) $$C_{S}\times P_{R}+C_{P}\times P_{L}+\text{CR}\times Q=\Delta \text{BV}+\sum_{n=1}^{3}C_{n}\times P_{e}+C_{4}\times P_{a}$$

where

$$\begin{array}{cc} C_{S}= & C_{1}+C_{4}+C_{5},\\ C_{P}= & C_{2}+C_{3},\\ \text{CR}= & C_{2}\times {\text{RV}}_{2}+C_{4}\times {\text{RV}}_{4}\times f_{4}+C_{5}\times {\text{RV}}_{5}\times f_{5},\\ f_{4}= & \frac{\text{TPR}}{{\text{RA}}_{4}+{\text{RV}}_{4},}\\ f_{5}= & 1-f_{4},\\ {\text{TPR}}^{-1}= & {\left({\text{RA}}_{4}+{\text{RV}}_{4}\right)}^{-1}+{\left({\text{RA}}_{5}+{\text{RV}}_{5}\right)}^{-1}. \end{array}$$

In the above equations, TPR represents the total peripheral resistance while *f*_4_ and *f*_5_ represent the fractional blood flow to the viscera and periphery, respectively.

In addition to the normal steady state depicted in Figure 1A, this model will be examined in two other steady states that demonstrate different points concerning model behavior. The first state, case 1, has already been discussed fully in the original published paper (Coleman et al. 1974), and is used here only to illustrate a point mentioned earlier about “movement of the conservation plane.” The second state, case 2, illustrates how it is possible to use this relatively simple model to gain insight into an observation made during human spaceflight.

**Figure 2:**
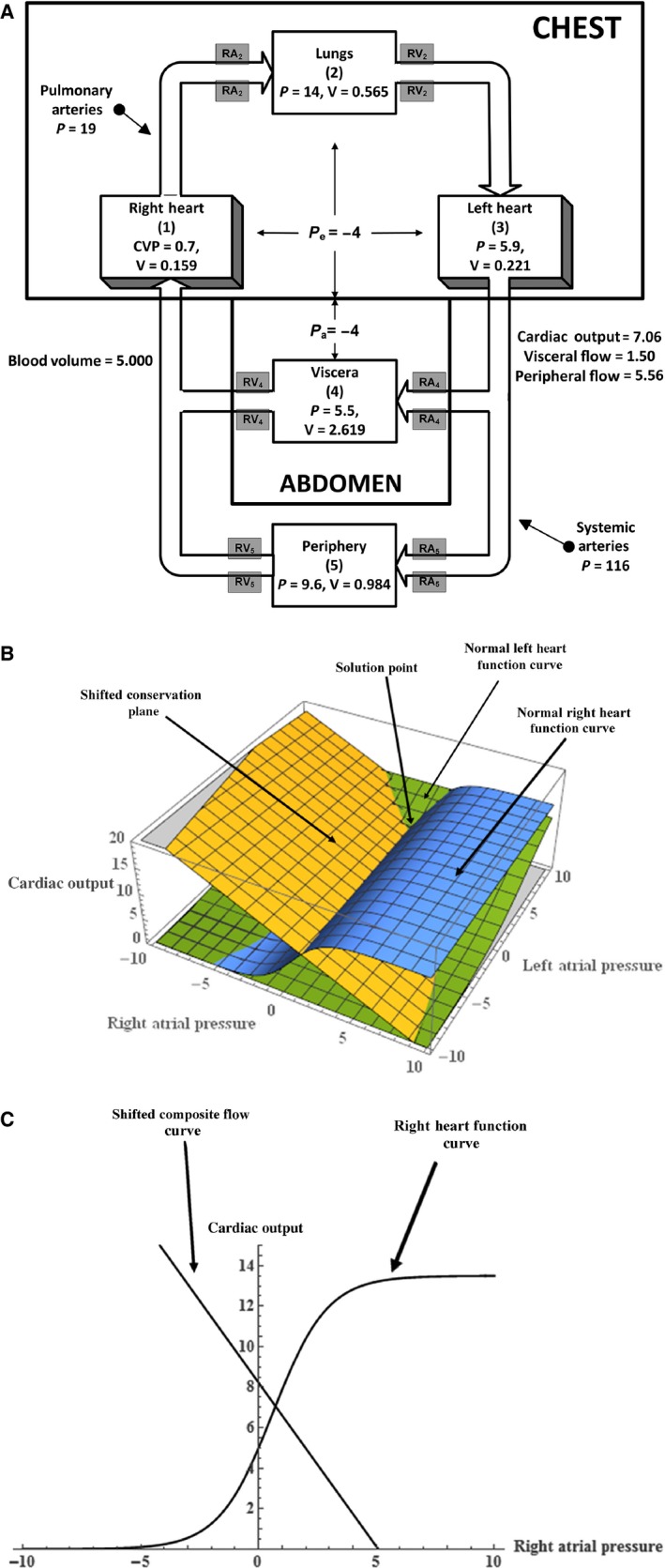
(A) The steady state of the five‐compartment model of the circulation with the peripheral arterial resistance reduced by 50% and the visceral arterial resistance doubled from the normal values. (See Case 1 in Table 2.) (B) Three‐dimensional graphical analysis of the model steady state with the peripheral arterial resistance reduced by 50% and the visceral arterial resistance doubled from the normal values. In this figure showing the intersection of the right and left Starling curves and the conservation plane, only the conservation plane has shifted from Figure 1B. (C) Two‐dimensional graphical analysis of the model steady state with the peripheral arterial resistance reduced by 50% and the visceral arterial resistance doubled from the normal values. In this figure showing the intersection of the right heart Starling curve and the composite flow (“venous return”) curve, only the latter has shifted from Figure 1C.

Case 1, whose steady state is depicted in Figure 2A, involves only changing two physiological parameters: the visceral arterial resistance, RA_4_, is doubled (from 36.8 to 73.6 mmHg/L/min), and the peripheral arterial resistance, RA_5_, is halved (from 38.6 to 19.2 mmHg/L/min). As can be seen from Figure 2A and Table 2, these physical changes have quite dramatic results, increasing the cardiac output by 42%, the arterial pressure by 15%, and moving nearly 80% of the cardiac output through the periphery. These results are in complete agreement with the original paper (Coleman et al. 1974), but are obtained with a different formalism. The original paper discusses a number of other studies and provides an excellent discussion of the utility of such a simple model.

**Figure 3:**
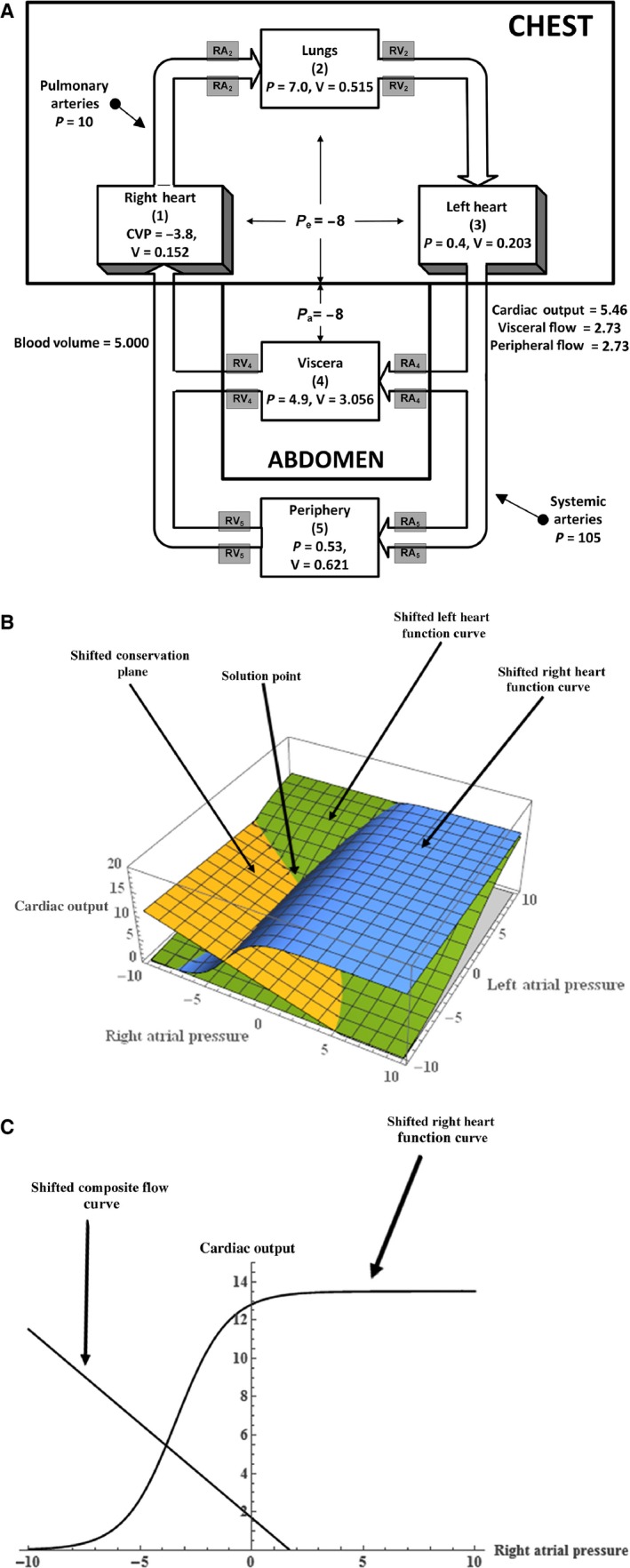
(A) The steady state of the five‐compartment model of the circulation with both the intrathoracic and abdominal pressures decreased from −4 to −8 mmHg. (See Case 2 in Table 2.) (B) Three‐dimensional graphical analysis of the model steady state with both the intrathoracic and abdominal pressures decreased from −4 to −8 mmHg. In this figure showing the intersection of the right and left Starling curves and the conservation plane, all three curves have shifted from normal. (C) Two‐dimensional graphical analysis of the model steady state with both the intrathoracic and abdominal pressures decreased from −4 to −8 mmHg. In this figure showing the intersection of the right heart Starling curve and the composite flow (“venous return”) curve, both curves have shifted from normal.

Case 2, whose steady state is depicted in Figure 3A, involves only reducing the intrathoracic (and abdominal) pressure from −4 to −8 mmHg. In this case, the resulting steady state involves a reduction of CVP (*P*_R_ in this model) by nearly 4 mmHg, but only modest increases in the cardiac output and the arterial pressure accompanied by a slight movement of blood into the chest compartments. This observation forms the cornerstone of a hypothesis related to the one of the early effects of microgravity on humans (White and Blomqvist 1998), a hypothesis that has stood the test of time.

### Graphical analysis of the five‐compartment model

It was mentioned in the general model description section that there were two graphical approaches to solve the resultant model equations. The first involves a *three‐dimensional graphical analysis*, and Figures 1B, 2B, and 3B illustrate the results of plotting the three relations defined by equations (1) and (15) using the (*P*_R_, *P*_L_, *Q*) coordinate system. These three equations are repeated here for reference:

(16) $$\begin{array}{cc} Q= & \frac{K}{1+\alpha_{R}\times \exp\left[-\beta_{R}\times \left(P_{R}-P_{e}\right)\right]},\\ Q= & \frac{K}{1+\alpha_{L}\times \exp\left[-\beta_{L}\times \left(P_{L}-P_{e}\right)\right]},\;\text{and}\\ Q= & \frac{\Delta \text{BV}+\sum_{n=1}^{3}C_{n}\times P_{e}+C_{4}\times P_{a}-C_{S}\times P_{R}-C_{P}\times P_{L}}{\text{CR}}. \end{array}$$

The solution, in all the three cases (normal, case 1, and case 2), is the value of *Q* at the mutual intersection of the three surfaces. Note that in case 1, the physiological parameters that change (resistances) are embedded in the “conservation plane” alone and this means that the new steady‐state value of *Q* results only from a shift of the conservation plane. In case 2, involving an intrathoracic pressure change, all of the three‐dimensional figures shift and the graphical result of Figure 3B show how these shifts lead to the new steady state.

The second graphical approach utilizes a *two‐dimensional analysis* resulting from plotting the linear relation described by equation (7) and the Starling function curve for the right heart using (*P*_R_, *Q*) coordinates. Figures 1C, 2C, and 3C illustrate the steady‐state results for the three cases (normal, case 1, and case 2). Note that in case 1, the arterial resistance changes only affect the “composite flow curve” defined by equation (7) since the Starling function curve is independent of resistance. This is analogous to the three‐dimensional shift of the conservation plane discussed above. In case 2, the intrathoracic pressure change affects both curves and Figure 3C shows how the resulting steady state is obtained.

### Single nonlinear equation analysis of the five‐compartment model

The single equation for this five‐compartment model has the same form as the general model equation for the special case where the two sides of the heart have the same maximum pumping capability and the same external pressure, equation (10). For convenience, the defining equations are repeated here:

(17) $$\begin{array}{cc} Z= & \mu +\lambda \times \log\left(\frac{1-Z}{Z}\right),\;\text{with}\\ \lambda = & \frac{\frac{C_{S}}{\beta_{R}}+\frac{C_{P}}{\beta_{L}}}{K\times \text{CR}},\;\text{and}\\ \mu = & \frac{\Delta \text{BV}+C_{4}\times P_{a}-\left(C_{4}+C_{5}\right)\times P_{e}-\log\left(\alpha_{R}^{\frac{C_{S}}{\beta_{R}}}\times \alpha_{L}^{\frac{C_{P}}{\beta_{L}}}\right)}{K\times \text{CR}} \end{array}$$

Using the normal model parameters from Table 1 and the altered parameters for case 1 (RA_4_ = 73.6, RA_5_ = 19.2 mmHg/L/min) and case 2 (*P*_e_ = *P*_a_ = −8 mmHg), one can easily compute the values shown in Table 1:

Normal State: *μ* = 0.326, *λ* = 0.0838, and *Z* = 0.370
Case 1: *μ* = 0.536, *λ* = 0.138, and *Z* = 0.523
Case 2: *μ* = 0.372, *λ* = 0.0838, and *Z* = 0.404

All of the physiological variables may be computed from these values of *Z* and they are presented in Table 2. As observed earlier, knowledge of only two “mathematical” parameters is sufficient to completely determine the solution to this steady‐state model with five compartments and over 20 physiological parameters.

The approach to solve the model equations using this single equation formulation is ideal if one wants to compute the values of the physiological variables, but the abstractness of the mathematical parameters does not lend itself to simple interpretation in the same way that the graphical approaches do. However, an advantage of the single‐equation formulation is the ease with which an exact sensitivity analysis may be carried out. As is well known, sensitivities for the model are related to the derivative ${\left(\partial v/\;\partial p\right)}_{0}$, where *v* is any variable, *p* is any parameter, and the subscript 0 indicates that the partial derivative is taken with all other independent parameters fixed. To compute sensitivities, it is convenient to use an equivalent form of equation (10):

(18) $$Q=\gamma +\omega \log\left(\frac{K-Q}{Q}\right)$$

where *γ* = *Kμ* and *ω* = *Kλ*. Then, differentiating term by term yields

(19) $${\left(\frac{\partial Q}{\partial p}\right)}_{0}=\frac{{\left(\frac{\partial \gamma }{\partial p}\right)}_{0}+\frac{Q-\gamma }{\omega }{\left(\frac{\partial \omega }{\partial p}\right)}_{0}}{1+\frac{K\omega }{Q(K-Q)}}$$

For example, if the parameter of interest is the intrathoracic pressure, *P*_e_,

(20) $${\left(\frac{\partial Q}{\partial P_{e}}\right)}_{0}=-\frac{C_{4}+C_{5}}{\text{CR}\left(1+\frac{K\omega }{Q(K-Q)}\right)}.$$

When the model is in the normal steady state, ${\left(\partial Q/\partial P_{e}\right)}_{0}=-0.485$ and the more usual sensitivity coefficient, defined as ${\left(\frac{\partial \log Q}{\partial \log P_{e}}\right)}_{0}$, has the value 0.388.

Sensitivities of other model variables or variables of interest may be computed directly from the cardiac output sensitivity and the equations presented in Table 2. For example, if arterial pressure, PA, is of interest, it follows that

(21) $${\left(\frac{\partial \text{PA}}{\partial P_{e}}\right)}_{0}=1-\frac{\left(C_{4}+C_{5}\right)\left(\text{TPR}+\frac{K}{\beta_{R}Q\left(K-Q\right)}\right)}{\text{CR}\left(1+\frac{K\omega }{Q(K-Q)}\right)}.$$

In the normal steady state, ${\left(\partial \text{PA}/\partial P_{e}\right)}_{0}=-8.89$ and ${\left(\frac{\partial \log\text{PA}}{\partial \log P_{e}}\right)}_{0}=0.356$

## Discussion and Conclusion

The major result presented in this paper is most clearly seen in the case where the right and left heart have the same maximum pumping capacity and the intrathoracic pressure is uniform. In that case, the resulting general equation for this class of steady‐state models of the circulation, equation (10), is a dimensionless, two‐parameter equation that holds for all models in this class, regardless of the number and arrangements of the compartments involved.

This result is not surprising. For many years, phenomenological models of the circulatory system have relied on this kind of simplification to support qualitative arguments concerning competing hypotheses. Although nearly every assumption made to develop this class of models is invalid, these models have at least one virtue other than simplicity; they are internally consistent. This fact allows them to be used to define experiments that can serve to test ideas emanating from the model whose data can lead to enhanced understanding of the system under investigation.

Although the final results obtained through this analysis are dependent of the specific form assumed for the Starling‐type cardiac function curves, equation (1), other forms for these cardiac function curves would yield slightly different but generally similar results. This can best be seen from the fact that the defining equation for the conservation plane is independent of the form assumed for the cardiac function curves, and the three‐dimensional graphical analysis would still yield a solution. The second and third approaches, a two‐dimensional graphical analysis, and a reduction to a single nonlinear equation are only dependent on the existence of an inverse to the cardiac function curves over the region of physiological interest, allowing *P*_R_ and *P*_L_ to be computed from *Q*. If this is the case, one can always reduce the analysis to either a two‐dimensional or a one‐dimensional problem, although the form of the result may be slightly different from that presented in this paper.

## Conflict of Interest

None declared.
